# Prevention of Canine Leishmaniosis in a Hyper-Endemic Area Using a Combination of 10% Imidacloprid/4.5% Flumethrin

**DOI:** 10.1371/journal.pone.0056374

**Published:** 2013-02-25

**Authors:** Domenico Otranto, Filipe Dantas-Torres, Donato de Caprariis, Giancarlo Di Paola, Viviana D. Tarallo, Maria S. Latrofa, Riccardo P. Lia, Giada Annoscia, Edward B. Breitshwerdt, Cinzia Cantacessi, Gioia Capelli, Dorothee Stanneck

**Affiliations:** 1 Dipartimento di Medicina Veterinaria, Università degli Studi di Bari, Valenzano, Italy; 2 Departamento de Imunologia, Centro de Pesquisas Aggeu Magalhães (Fiocruz-PE), Recife, Pernambuco, Brazil; 3 Intracellular Pathogens Research Laboratory, College of Veterinary Medicine, North Carolina State University, Raleigh, North Carolina, United States of America; 4 Center for Biodiscovery and Molecular Development of Therapeutics, James Cook University, Cairns, Australia; 5 Istituto Zooprofilattico Sperimentale delle Venezie, Laboratory of Parasitology, Legnaro, Italy; 6 Bayer Animal Health GmbH, Leverkusen, Germany; University of Melbourne, Australia

## Abstract

**Background:**

Dogs are the main reservoir hosts of *Leishmania infantum*, the agent of human zoonotic visceral leishmaniosis. This study investigated the efficacy of a polymer matrix collar containing a combination of 10% imidacloprid and 4.5% flumethrin as a novel prophylactic measure to prevent *L. infantum* infections in young dogs from a hyper-endemic area of southern Italy, with a view towards enhancing current control strategies against both human and canine leishmaniosis.

**Methodology/Principal Findings:**

The study was carried out on 124 young dogs, of which 63 were collared (Group A) while 61 were left untreated (Group B), from March-April 2011 until March 2012. Blood and skin samples were collected at baseline (April 2011) and at the first, second, third and fourth follow-up time points (July, September 2011 and November 2011, and March 2012, respectively). Bone marrow and conjunctiva were sampled at baseline and at the fourth follow-up. Serological, cytological and molecular tests were performed to detect the presence of *L. infantum* in the different tissues collected. At the end of the trial, no dog from Group A proved positive for *L. infantum* at any follow-up, whereas 22 dogs from Group B were infected (incidence density rate = 45.1%); therefore, the combination of 10% imidacloprid and 4.5% flumethrin was 100% efficacious for the prevention of *L. infantum* infection in young dogs prior to their first exposure to the parasite in a hyper-endemic area for CanL.

**Conclusions:**

The use of collars containing 10% imidacloprid and 4.5% flumethrin conferred long-term protection against infection by *L. infantum* to dogs located in a hyper-endemic area, thus representing a reliable and sustainable strategy to decrease the frequency and spread of this disease among the canine population which will ultimately result in the reduction of associated risks to human health.

## Introduction

*Leishmania infantum* is a major protozoan parasite transmitted by arthropod vectors causing visceral and cutaneous leishmaniosis in dogs and humans in southern Europe, Africa, Middle and Far Eastern countries and Central and South America, with approximately 500,000 new infections recorded each year [1], [2]. In southern Europe (including Turkey), leishmaniosis caused by *L. infantum* is endemic, with a total of 3,950 new human cases reported each year [3]. Dogs play a major role as the main reservoir hosts of zoonotic visceral leishmaniosis [4]. Indeed, canine leishmaniosis (CanL) is amongst the most widespread vector-borne parasitic diseases affecting dogs from all continents, except Oceania [5]. In areas where the competent vectors, i.e. phlebotomine sand flies of the genus *Phlebotomus* (in the Old World) and *Lutzomyia* (in the Americas), are widespread, human and canine infections are closely associated [6], [7] and ‘hotspots’ of infection correspond to areas whose environmental and climatic conditions are ideal for the development of the arthropod vectors [8], [9]. Therefore, over the last decades, considerable efforts have been directed towards monitoring the prevalence and incidence of CanL in both endemic and non-endemic areas, as well as developing novel and cost-effective control strategies against this devastating disease. Recent studies have reported the diffusion of CanL by *L. infantum* in previously non-endemic areas (e.g., from northern Argentina to northern United States and some provinces of southern Canada) [4], [10]. Also, the disease has spread from southern Mediterranean regions to northern Europe [11], [12]. CanL is highly prevalent in dogs in South America and in the Mediterranean regions, with up to 60% of infected dogs being asymptomatic, making current estimations of prevalence of infection based on the detection of clinical signs unreliable in both non-endemic and hyper-endemic areas [13], [14].

As a consequence of the eco-epidemiological complexities involved in the route of transmission of *L. infantum*, the control of CanL has proven challenging, and none of the proposed strategies (e.g., dog culling in Brazil, environmental spraying with insecticides) have yielded satisfactory results thus far [4]. For instance, the use of dichlorodiphenyltrichloroethane (DDT) house spraying against phlebotomine sand flies in Brazil has been unsuccessful [15], probably due to failure to apply the insecticide at the right time of year [16] and to the impracticability to reach the natural breeding sites of these insects [17]. Also, due to environmental side effects and human health hazards, organochlorides [e.g., DDT and benzene hexachloride (BHC)] have gradually been substituted by synthetic pyrethroids [16]–[18]. The use of pyrethroids with repellent properties in spot-on formulations [19] or as impregnated collars [20] has represented a useful and cost-effective approach to reduce the risk of *L. infantum* infection in dogs in endemic areas. In a previous study [21], the use of deltamethrin-impregnated dog collars resulted in a protection rate against canine infections by *L. infantum* of 50% and 86%, respectively, over two consecutive transmission seasons in Europe [22]. A combination of imidacloprid 10% and permethrin 50% in a spot-on formulation was also effective in reducing *L. infantum* infection in large populations of dogs in kennels [19], [23], as well as of a range of canine tick-borne diseases (e.g., babesiosis and canine cyclic thromobocytopenia caused by *Babesia canis* and *Anaplasma platys*, respectively), both in autochthonous animals and naïve beagle dogs experimentally introduced into a kennel environment together with native, tick-infested dogs [24].

Recently, a polymer matrix collar containing a combination of 10% imidacloprid and 4.5% flumethrin (Seresto®, Bayer Animal Health), hereinafter referred as “collars”, with both repellent and insecticidal properties against fleas and ticks, has been developed for use in dogs and cats [25],[26]. Previous efficacy studies have demonstrated protection against tick and flea infestations over a period of eight months, which has been attributed both to the slow-release collar matrix system and to the synergistic action between the pyrethroid flumethrin and the neonicotinoid imidacloprid [25], [26]. Despite these promising results, no data describing the repellent activity of this collar formulation against phlebotomine sand flies or its efficacy in preventing CanL in endemic areas is available. In the present study, we addressed this gap in knowledge by investigating the efficacy of this collar for the prevention of infection by *L. infantum* in young dogs living in a hyper-endemic area of southern Italy.

## Materials and Methods

### Ethical statement

The study was conducted according to the principles of Good Clinical Practice (VICH GL9 GCP, 2000 http://www.emea.eu.int/pdfs/vet/vich/059598en.pdf) in the guideline for the testing and evaluation of the efficacy of antiparasitic substances for the treatment and prevention of tick and flea infestation in dogs and cats (EMEA/CVMP/005/00, 2000 http://www.emea.eu.int/pdfs/vet/ewp/000500en.pdfl) and the guideline on Statistical Principles for Veterinary Clinical Trials (CVMP/816/00, 2000 www.emea.eu.int/pdfs/vet/ewp/081600en.pdf). The study design and the experimental procedures were approved and authorized by the Italian Ministry of Health (authorization number DGSA n°. 0001997; 04/02/2011).

### Study area

The trial was conducted from March 2011 to October 2012 in a private animal shelter in Putignano, province of Bari, Apulia region, Italy (latitude 40°51 N, longitude 17°7 E, altitude 372 m above sea level). The shelter is managed by a private citizen association. The occurrence of sand flies at the study site had been monitored over the previous three years [27]; a year crude incidence of 47.6% for *L. infantum* infection in the native canine population had been estimated over the previous two years [19]. Approximately 200 dogs, not enrolled in the study protocol, were allowed to roam freely in the surroundings, with the purpose of maintaining the natural conditions of infection existing within the study site.

### Study design and experimental procedures

This field study was designed to evaluate the efficacy of collars for the prevention of *L. infantum* infection in dogs. On the enrolment day, one collar was applied around the neck of each dog of Group A and its length adapted to the size of the dog with a ratchet closure mechanism. Overlapping ends were cut off to accommodate the required length and to avoid other dogs chewing at a lose end. Dogs <6 months of age were assigned by coin toss to two different Groups: Group A – dogs treated with the collars on day 0 and remaining under treatment until March 2012; Group B – untreated control dogs. The homogeneity of the two Groups in relation to dogs' epidemiological data (i.e., sex, weight and coat length) was evaluated using the chi-square test and one-way ANOVA on the enrolment day. Collars were applied to dogs from Group A according to their body weight (collar dose ranges: ≤8 kg: small collar; >8 kg: large collar). Throughout the study and where applicable, collars were replaced according to the increasing body weight of growing dogs. Any collar losses/removals were recorded and lost/removed collars were reapplied immediately; any irreparably damaged collar was replaced by a new one, within two days.

In accordance with the EMEA/CVMP/005/00-Rev2 guidelines, each dog was individually identified using microchips and photographed. Due to the visibility of the collars, personnel involved in the direct handling of animals were not blinded as to treatment or control Group designation; nevertheless, the laboratory personnel were not informed of the composition of the study Groups and Group designation was not indicated on sample tubes and slides.

At their enrolment, from March to April 2011 (baseline), all data associated with each dog (i.e., sex, age, weight, and coat length) was recorded on individual forms. Each dog was clinically examined and serum, blood, skin, bone marrow and conjunctival swab samples were collected for testing. At the first, second and third follow-up times (July, September and November 2011, respectively) and at fourth follow-up time point (March 2012, final test) skin biopsies and conjunctival swabs were collected from all dogs included in the trial, whereas the bone marrow was sampled at the baseline and at the fourth follow-up time point (Table 1). Dogs were examined clinically every ten days throughout the study and at each follow-up time point. Clinical signs suggestive of CanL (e.g., ocular lesions, skin lesions, weight loss, and lymphadenomegaly) were reported in each dog's individual medical record.

**Table 1:** Incidence density rate (IDR) of leishmaniosis in dogs from the collared (A) and uncollared control (B) Groups.

Dogs enrolled	Sampling date	Number of dogs in the cohort		Number of new cases^a^		Dog-months of follow-up		Incidence Density rate/year (95% CI)	
		A	B	A	B	A	B	A	B
Baseline	March–April 2011	63	61	-		-	-	-	-
Follow-up 1	July 2011	63	61	0	0	175.8	136.6	0	0
Follow-up 2	September 2011	62	53	0	1	163.1	140.3	0	8.55 (1.02–16.08)
Follow-up 3	November 2011	62	52	0	10	127.7	107.1	0	100
Follow-up 4- final test	March 2012	62	51	0	10	251.1	174.4	0	68.82 (56.08–81.52)
Total				0	21	717.6	558.4	0	45.13 (31.44–58.76)

a = dogs positive by one or more parasitological, serological and/or PCR tests.

In March 2012, at the end of the treatment period, dogs from both Group A and B that were not adopted by private citizens remained untreated until October 2012, thereby facilitating exposure to *L. infantum* throughout the following sand fly season. In October 2012, a complete sample collection was performed on the remaining 111 animals (62 from Group A and 49 from Group B, respectively). This follow-up testing, including serological, cytological and PCR, was performed seven months after the collars were removed (March 2012) to determine the incidence of CanL amongst dogs either collared (Group A) or not collared (Group B) over the previous year, and left untreated throughout the following sand fly season (May–October 2012).

### Experimental animals, housing and sample size

Of the 176 young dogs enrolled in the study, 52 (29.5%) died within the first six weeks as a consequence of an outbreak of canine parvovirosis (data not shown). The remaining 124 dogs of both sexes, including several breeds, <6 months of age and housed in the animal shelter were included. All dogs were in acceptable health conditions, which fulfilled the veterinary inclusion criteria at baseline, start day 0 (SD 0). Each dog was vaccinated by two intramuscular injections (at a two-week interval) of Duramune® DAPPI+LC (Fort Dodge Animal Health, Italy) against common dog pathogens (canine parvovirus, adenovirus type 2, distemper virus, *Leptospira canicola* and *Leptospira icterohaemorrhagiae*) and each dog was dewormed using a combination of febantel/pyrantel/praziquantel (Drontal plus®; Bayer AG, Germany) following the manufacturers' instructions.

Dogs were kept in wire mesh cages (n = 21) of approximately 10 m×20 m located near stonewalls, known to represent natural resting sites for sand flies. Fences separating the cages of the treated and untreated Groups were at least 2 m apart. All dogs were maintained under the same housing conditions before, during, and after the study and fed commercial food once per day, while water was provided *ad libitum*. Dogs were removed from the study only if their general health deteriorated, if skin lesions related to the product application were observed, or if they died. The use of other ectoparasiticides on the study animals or in the environment was not allowed throughout the study period, except when circumstances (e.g., heavy tick or flea infestations) represented a threat to the dogs' welfare.

### Diagnostic procedures

Blood samples (2 ml) were collected from the brachial or jugular veins and transported to the Faculty of Veterinary Medicine of the University of Bari (Italy) in ethylene-diamine- tetraacetic acid vials (EDTA). On the same day, samples were centrifuged at 1,678 *g* for 10 minutes, and sera were separated and stored in individually labelled Eppendorf tubes at −20°C until tested. Bone marrow samples were obtained by aspiration from the iliac crest using Rosenthal needles (16 or 18 gauges) and stored at −20°C in individual Eppendorf tubes with 1 ml of phosphate buffer saline (PBS) until molecular processing. In addition, bone marrow samples were smeared on slides for cytological examination in an effort to detect *L. infantum* amastigotes. Skin tissue samples of approximately 0.5 cm^2^ were collected from the intrascapular region and processed as described previously [19], [23]. The conjunctival swabs were transferred to tubes containing physiological saline solution until the arrival to the laboratory and stored at −20°C.

An indirect immunofluorescent antibody test (IFAT) was performed using promastigotes of *L. infantum* zymodeme MON1 as antigen as described elsewhere [19]. Samples were scored positive when a clear cytoplasmatic or membrane fluorescence with promastigotes could be observed using a cut-off dilution of 1∶80; positive sera were titrated until negative. The cytological preparations of bone marrow smears were microscopically examined by staining with MGG Quick Stain (Bio Optica, Italy) in order to detect the presence of amastigotes.

PCR for the amplification of *Leishmania* DNA was performed on bone marrow, conjunctival swab and skin samples. Total DNA was extracted using the QIAampDNA Micro Kit (Qiagen, GmbH, Hilden, Germany) and the Genomic DNA Purification Kit (Gentra Systems, Minnesota, USA), respectively, and a fragment of *L. infantum* kinetoplast DNA minicircle was amplified using the MC1/MC2 primer set [14]. Amplicons were resolved in ethidium bromide-stained (2%) agarose gels (Gellyphor, Italy) and sized by comparison with markers in the Gene Ruler™ 100 bp DNA Ladder (MBI Fermentas, Lithuania). Gels were photographed using a digital documentation system (Gel Doc 2000, BioRad, UK).

### Environmental examination procedures

From May to October 2011 and 2012, the density of the sand fly population was assessed using castor oil-coated sticky traps (30 cm×21 cm) for 24 hrs periods. Sand flies were collected twice a month after their first appearance and then every 3 weeks until the collection of the last insect. Sand flies were counted and identified at the species level according to Maroli et al. [28]. The values of mean temperature and relative humidity were recorded throughout the study period by a data logger (HD 206 Delta OHM, Padova, Italy) every 30 min for a total of 48 records per day. Then, the mean temperature (°C) and relative humidity (%) recorded during the whole day (24 h) and night (9:00 pm to 4:00 am) were calculated.

### Statistical analysis

The minimum sample size (n = 49) was calculated using the software WinEpi (http://www.winepi.net/uk/index.htm) to estimate differences between proportions (i.e., incidence) from two populations and with the following assumptions: (i) expected proportion in Group A (i.e., treated animals) = 5%; (ii) expected incidence in Group B (untreated animals) = 15%; (iii) power = 85%; (iv) level of confidence = 95%. To overcome the potential loss of animals throughout the study, >60 instead of 49 dogs were enrolled in each Group (63 in Group A and 61 in Group B, respectively). In addition, the incidence of *L. infantum* infections was determined by means of incidence density rate (IDR) [23], [29], calculated as follows: IDRs = number of *L. infantum* positive dogs/number of dog-months of follow-up (i.e., the number of months between the previous and the following assessment for each dog at risk for *L. infantum* infection). Differences between incidence rates in Groups A and B were calculated using the Yates corrected ÷2 test. Dogs tested once (e.g., lost, dead) did not contribute to the calculation of incidence, whereas dogs sampled at least at two time points contributed to the IDR calculation with the number of months for which they remained in the study. The year crude incidence (calculated considering the test results at the end of the study) was also calculated.

The analysis of efficacy of the collar in preventing CanL was based on the population, which reasonably matched the protocol, and was evaluated based on the protection of the animal from *L. infantum* infection during the time at risk. Infection was diagnosed at each follow-up and calculated using the following formula: % of protection = (% of animals positive in control Group - % of animals positive in treated Group/% of animals positive in control Group)×100. For this study, a “positive dog” was defined as any dog that was cytology-, serology- (antibody titre >1∶80) or PCR-positive for *L. infantum* in one or more than one diagnostic test, thus including all dogs with evidence to support exposure to sand flies which resulted in transmission of *L. infantum*, regardless of whether the infection progressed to disease.

## Results

The collared (n = 63; Group A) and control (n = 61; Group B) animals were homogeneous (p>0.05) in terms of sex, age, weight and coat length. Following enrolment in the study, 9 (7.2%) dogs died soon after the second follow-up time point (i.e., one from Group A, and 8 from Group B), while two dogs from Group B died by the third and fourth follow-up time points, respectively. Of the 124 dogs initially assessed by serological, parasitological and molecular tests only one (0.06%) was *L. infantum*-positive (based on PCR amplification of parasite DNA from a skin sample). This dog (Group A) remained in the same enclosure together with three other dogs of the same Group, albeit it was not considered in the calculation of incidence.

The IDRs for each Group at the successive follow-up time points are shown in Table 1. No dogs in Group A became positive for *L. infantum*, whereas *Leishmania* infection, inferred by positivity in at least one of the three tests performed (i.e., serology, cytology and PCR) at the first, second, third and final follow-up time points was diagnosed in 21 (IDR = 45.1%) dogs in Group B. Of the 51 dogs in Group B that were available at the final follow-up time point, 18 were *L. infantum*-positive, thus resulting in a year crude incidence of 35.3%. Results of serology, cytology and PCR on different tissues for each positive dog are reported in Table 2. No dogs were positive at the first follow-up (i.e., June) and only one dog was positive at the second follow-up (September 2011); at the third follow-up (November 2011), ten dogs had become serologically positive. Of these, seven were also skin-PCR positive. Twenty of 21 dogs (cf. Table 2) remained positive at one or more testing modalities, at the fourth (final) follow-up time point. All of these dogs were seropositive, nine dogs were bone marrow-PCR positive and, of these, six were also skin-PCR positive; amastigotes were detected at the cytological examination of the bone marrow from three dogs. All dogs positive at the PCR performed on the conjunctival swab, but one, were also bone marrow-PCR positive, thus indicating a disseminated status of the infection. Antibody titres, at the final follow-up time point, were high (ranging from 1∶160 up to 1∶1.240) in all dogs that were bone marrow-PCR positive. In addition, dog 17 had an antibody titre of 1∶1.240 and displayed positivity at cytology and PCR of both skin and conjunctiva (Table 2).

**Table 2:** Results at IFAT (the highest dilution titre is reported), cytological examination and PCR results for skin, bone marrow and conjunctival swabs of positive dogs at follow-up time points and the final test period.

Dogs	September 2011	November 2011		March 2012				
	Serology	Serology	PCR	Cytology	Serology	PCR		
			Skin			Skin	Bone marrow	Conjunctival swabs
Dog1	−	−	−	−	−	+	−	−
Dog 2*	−	−	−	+	1∶320	+	+	−
Dog 3	−	−	−	−	−	+	−	−
Dog 4	−	−	−	−	1∶160	+	+	−
Dog 5	−	1∶80	+	−	1∶160	−	−	−
Dog 6	−	−	−	+	1∶320	+	+	−
Dog 7	−	1∶80	−	+	1∶80	−	−	−
Dog 8*	−	1∶80	+	+	1∶160	−	−	+
Dog 9	−	−	−	−	1∶160	+	−	−
Dog 10	−	−	−	−	1∶320	−	+	−
Dog 11	−	1∶80	+	−	1∶640	−	+	+
Dog 12	−	1∶80	+	−	1∶80	−	−	−
Dog 13	−	1∶80	−	−	−	−	−	−
Dog 14	−	1∶80	+					
Dog 15	−	−	−	−	1∶160	+	+	+
Dog 16	−	−	−	−	−	+	−	+
Dog 17*	−	1∶80	+	+	1∶1240	+	+	+
Dog 18*	−	1∶80	+	−	1∶160	+	+	+
Dog 19*	−	+	−	−	1∶320	−	+	+
Dog 20	+	−	−	−	−	−	−	−
Dog 21	−	−	−	−	1∶80	+	−	−

Animals displaying clinicopathological alterations suggestive for *Leishmania infantum* infection are marked with an asterisk.

**Figure 1:**
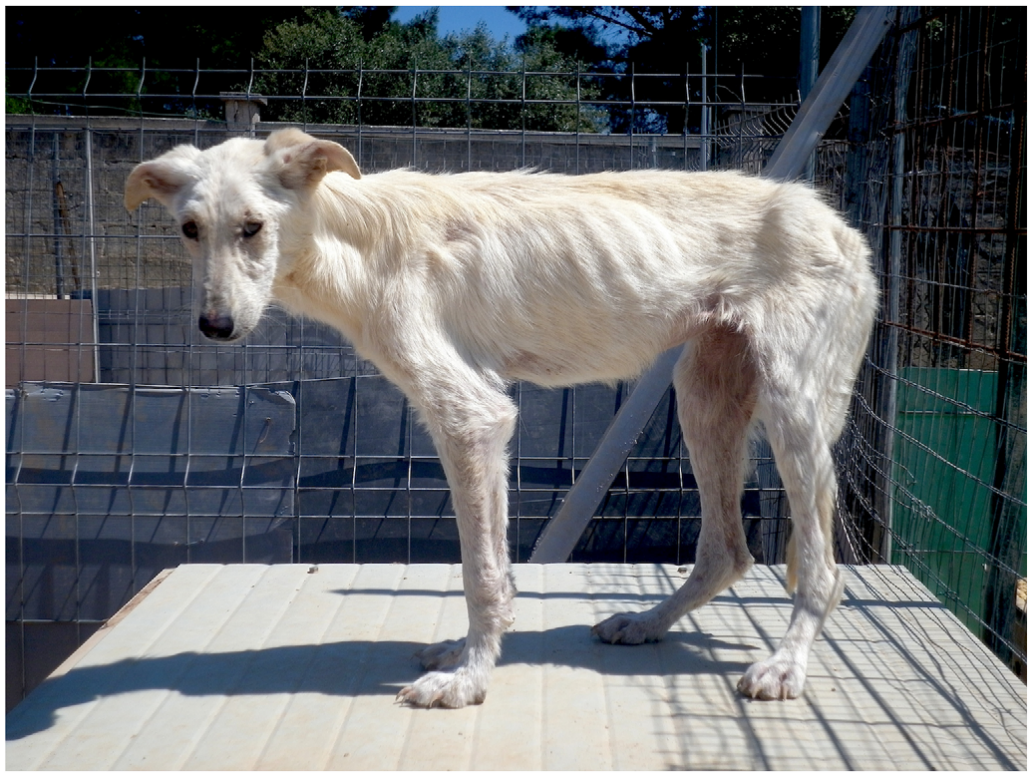
A dog from Group B displaying clinical signs of canine leishmaniosis.

Five (27.8%) out of the 18 dogs that were positive at serology and two additional diagnostic tests (Table 2) displayed clinical signs (i.e., lymphadenomegaly, furfuraceous dermatitis, onychogryphosis, depression and weight loss, diarrhoea) on April 2012 (Figure 1) with haematological abnormalities mainly consisting of anaemia, leukocytosis, neutrophilia with left shift, lymphocytosis, monocytosis, eosinophilia, thrombocytopenia, hypoalbuminemia and hypergammaglobulinemia. No dogs from Group A tested *Leishmania*-positive during the study leading to a final protection efficacy of 100% in collared animals.

Collars were replaced on young dogs at different times (i.e., 8 within 3 months; 35 within 6 months and 20 within 8 months). While the replacement was mostly due to the increasing bodyweight (n = 46; 73%), in some cases (n = 17; 27%) a second collar replacement was necessary due to loss of collars during fights or play. Four dogs needed a third collar replacement within 1.3 months from the second replacement and two of them repeatedly destroyed the collar thus needing a fourth (within 1.3 months) and a fifth (within 1.1 months) replacement. Of these, one dog needed two additional collar replacements (2.3 and 2.1 months apart, respectively). Due to the high tick pressure in the environment and on animals in Group B, an environmental treatment outside the enclosures hosting Group B dogs with Bayticol (Flumetrine) and an ‘on-animal’ treatment with Frontline Combo (Fipronil and methoprene) were authorised twice in June 2011 and August 2012, respectively.

Following the end of the treatment period (March 2012), 111 animals (62 from Group A and 49 Group B) remained untreated throughout the following sand fly season. In October 2012, out of the 111 dogs, in Group A 8/62 (12.9%) and in Group B 29/49 (59.2%) tested *L. infantum*-positive, leading to an overall 2012-year incidence of 20.6% (19 new cases out of 92 negative dogs in March 2012) for *L. infantum*, seven months after the collars were removed.

In both years phlebotomine sand flies first appeared during the last week of May when the temperature increased and the relative humidity decreased with most of the sand fly specimens collected in July. The last collection of sand flies occurred in mid October 2011 and at end of September 2012. Throughout both years, *Sergentomya minuta* (66.6%) was the most frequently identified species, followed by *Phlebotomus perniciosus* (15.1%), *Phlebotomus neglectus* (8.8%) and *Phlebotomus papatasi* (0.23%). The remaining specimens were identified as *Phlebotomus* spp. (9.3%) due to their deteriorated conditions. The majority of *P. perniciosus* specimens were collected on August and July 2011 and 2012, respectively, when the highest monthly mean temperature and a low monthly mean relative humidity were recorded.

## Discussion

The present study reports the results of the first large-scale investigation of the efficacy of a combination of 10% imidacloprid and 4.5% flumethrin in a collar formulation for the prevention of one of the most important arthropod-borne, zoonotic diseases of dogs worldwide. This combination proved safe and 100% efficacious in preventing *L. infantum* infection in young dogs after their first exposure to the aetiological agent of CanL in a hyper-endemic area. Dogs were treated according to their increasing bodyweight and collars were replaced, if needed, due to animal growth, collar loss or end of the 8 months label efficacy period. The wearing time of a single collar ranged from a minimum of 3 months (n = 8 individuals) to up to 6 (n = 35) and even 8 (n = 20) months. Accordingly, in 87.3% (n = 55) of the collared animals the collar's protective efficacy against *L. infantum* was investigated throughout a period of 6–8 months. Based on current knowledge of sand fly seasonal dynamics [27], as well as data shown herein, the duration of protection conferred by a single collar spans an entire sand fly season in southern Italy. The prolonged efficacy of the 10% imidacloprid and 4.5% flumethrin combination is most likely linked to the fact that the collar matrix allows an even release of the active ingredients at a constant concentration [16], [25], [26], unlike the registered deltamethrin collars [20], [21], [30].

Whilst no collared dogs (Group A) were infected by *L. infantum*, IDRs as high as 45.1% were recorded in dogs left uncollared (Group B). This high incidence differs from previous observations conducted in same area, being approximately 3-fold higher than the incidence previously recorded in owned (9.5%) [31] and kennelled dogs (13.6%) [23], respectively. However, the incidence of *L. infantum* infection as detected by parasitological, serological, and molecular techniques, was similar to that recorded in two earlier studies conducted in the same kennel (IDR = 44.4%) [19], which confirms the persistent sand fly activity at this site. The high endemicity among the canine population of this area might also be inferred by the occurrence of signs indicative of leishmaniosis (i.e., furfuraceous dermatitis, onychogryphosis, lethargy, poor body condition, anorexia and lymphadenomegaly) in five out of 18 (27.7%) dogs in Group B after exposure to sand flies during only one vector season (March 2012). These dogs also displayed laboratory abnormalities compatible with CanL (i.e., anaemia, eosinophilia, thrombocytopenia, hypoalbuminemia and hypergammaglobulinemia, and in two cases azotemia). No collared dog seroconverted, became PCR-positive or displayed clinical signs indicative of CanL, which can be attributed to the anti-feeding activity of flumethrin. Indeed, this compound is known to reduce the number of insect bites and, in turn, the infection challenge, thereby preventing a shift towards a non-protective immune response and the development of disease [32].

The overall protection rate against CanL was higher than that calculated in studies conducted using deltamethrin collars (maximum 86%) in southern Italy [14], [24], [30], [33], where post-exposure infection incidences up to 91.7% had been recorded [34]. The safety of the collar evaluated in this study was also assessed; the integral safety-closure ratchet mechanism of the collar proved pivotal in ensuring the safety of the product, especially in young gang-housed kennel dogs living in the same enclosure, by allowing the collar to be widened by the distance to the next rib [25], [26] thus preventing accidents during playing or fighting. The number of collar losses recorded (27%) could be associated to the high activity and playing attitude of young animals, as well as with the first heat of females, and mating-related fighting between males in the same confined environment. Two dogs repeatedly destroyed the collar, requiring up to seven replacements. Overall, even in presence of high animal density in a confined environment, the collar loss rate was lower than that recorded in previous studies conducted on owned dogs (up to 35%) [30].

The ecological aspects of the sand fly populations over the two years were similar to those observed in previous studies conducted at the same site [19], [27], with *P. perniciosus* and *P. neglectus* being the most represented species. The relatively low number of sand flies collected during the first and second year of the study might relate to environmental treatments with Bayticol (Flumethrine), which was carried out on June 2011 and August 2012 to reduce heavy tick infestations. Nonetheless, the constant occurrence of sand flies is indicative of the real risk of infection by *L. infantum* in dogs and humans in this area. The dog shelter chosen for this survey is located about 200 m from the nearest households in Putignano. A green area with Mediterranean vegetation and olive and cherry fields surrounds the kennel, which is delimitated by stonewalls that represent typical resting sites for sand flies. The occurrence of five species of phlebotomine sand flies (i.e., *P. perniciosus*, *P. neglectus*, *P. papatasi* and *S. minuta*) collected in this study, confirms the results of previous entomological surveys conducted in southern Italy [27], and indicates a high degree of species richness in the populations of these arthropod vectors in this urban area. Under the above circumstances, the enrolment of dogs housed in kennels in an hyper-endemic area (rather than privately owned dogs) is of relevance, considering that the large number of animals positive for *L. infantum* acts as a natural source of infection for sand flies in that specific location and reduces bias such as a lack of control over decisions made by individual owners, different management practices (e.g., dogs kept indoors overnight), risk of treatment of animals and/or environment with ectoparasiticides. Confined dogs were also subjected to the same sand fly biting pressure, which is guaranteed by the same sand fly microhabitats and a similar sand fly burden.

As a consequence of the multiple factors that determine whether and how rapidly there is progression to leishmaniosis after *L. infantum* infection in individual dogs (e.g., immune response, level of exposure and repeated transmission by infected sand fly bites), the “positive” status of each dog in this study was established using a combination of results from serological (IFAT), parasitological (cytology) and molecular (PCR) tests performed on different sample sources (i.e., bone marrow, buffy coat, conjunctival swab, and skin). With the exception of one dog (dog 20), uncollared positive dogs were detected for the first time in November 2011 by serological testing (n = 10) and PCR on skin samples, thus indicating that all dogs were exposed to *L. infantum* during the first sand fly season. The progression from infection to clinical disease was confirmed by the fact that all these dogs except one remained seropositive and bone marrow-PCR positive at the subsequent follow-up time points, and nine out of 21 dogs were also cytologically positive based on amastigote detection. Serology failed to detect infection in six out of 21 positive dogs that were cytology- or PCR-positive, thus indicating that the former should be used in combination with other diagnostic tests to assess the infection status of dogs exposed to sand flies in endemic regions. This finding is most likely related to the fact that dogs infected by *L. infantum* (especially if asymptomatic) may not seroconvert immediately, as the incubation period prior to seroconversion may range from 3 months to 7 years [35]. Thus, when considered alone, seronegativity may lead to false-negative results in control and study Groups. In this study, detection of the parasites (cytology) and/or seropositivity was associated with clinical signs only in 27.7% of *L. infantum* infected dogs, thus confirming the results of previous studies which indicated that a large proportion of infected dogs remain asymptomatic for prolonged periods [14]. Based on this knowledge, previous studies that solely employed serological techniques for inferring the efficacy of insecticides for the prevention of *L. infantum* infections [30], [33] should be considered with caution.

## Concluding Remarks

While the activity of sand flies in southern Europe peaks during summer (i.e., June, July and August), the risk of sand fly bites spans from late spring to autumn, which coincides with the peak of tourism in these areas, therefore maximising the risk of acquiring canine and human leishmaniosis, with the subsequent introduction of infected dogs into non-endemic areas [36]. Therefore, the protection of canine populations that may act as an important reservoir of *L. infantum* infection is crucial (at least) between May and October. The results from the present study indicate that the use of 10% imidacloprid and 4.5% flumethrin in collar formulation offers long-term (i.e., up to 8 months), reliable and sustainable protection against *L. infantum* infection in hyper-endemic areas. The widespread use of this effective prophylactic measure, combined with additional control strategies directed towards the reduction of the *L. infantum* infection in the sand fly populations in the same areas will ultimately assist in the elimination of the risks for the canine and human populations alike. As previously discussed, a “One Health” approach to the control of leishmaniosis is critically needed [4].
